# Trends in melanoma incidence at Hospital Italiano de Buenos Aires, 2007–2016^☆^

**DOI:** 10.1016/j.abd.2020.10.013

**Published:** 2021-11-17

**Authors:** Adriana Raquel Rinflerch, Victoria Ines Volonteri, María Cecilia Roude, Laura Vanina Pagotto, Melina Pol, Luis Daniel Mazzuoccolo

**Affiliations:** a Instituto de Biología Subtropical CONICET, Universidad Nacional de Misiones, Misiones, Argentina; b Servicio de Dermatología, Hospital Italiano de Buenos Aires, Buenos Aires, Argentina; c Servicio Anatomía Patológica del Hospital Italiano de Buenos Aires, Buenos Aires, Argentina; d Departamento de Investigación, Hospital Italiano de Buenos Aires, Buenos Aires, Argentina; e Servicio Anatomía Patológica, Hospital Italiano de Buenos Aires, Buenos Aires, Argentina

Dear Editor,

There are three types of melanomas: uveal, mucosal, and cutaneous. Cutaneous melanoma is the most common subtype, and it causes most skin cancer deaths.^1^ Despite the fact that the risk factors for melanoma are known today and there are social prevention and advertising campaigns about skincare, disease rates have increased worldwide in recent years.^2^ The demographic distribution of melanoma incidence is directly related to environmental and genetic factors such as geographic sun intensity, and skin phototypes of the population. Immunosuppression and numerous episodes of sunburn increase the risk even more.^3^

The Hospital Italiano de Buenos Aires is a private health care institution where approximately 300,000 people from Buenos Aires City (BA) are treated annually. Half of them are members of the prepaid Plan de Salud del Hospital Italiano de Buenos Aires (Medical Care Program – HMCP).^4^ As little is known about melanoma incidence either in the BA or the Argentinean populations, we sought to explore that incidence in our HMCP population.

The Hospital Italiano de Buenos Aires Clinical Research and Bioethics Committee approved this study.

Rates were reported with the corresponding 95% Confidence Intervals (CIs). The differences were considered significant when the p-value was less than 0.05; STATA software (Stata Corp LLC, TX; version 14.2) was used for calculations.

A retrospective cohort study was carried out, including a population with reports of invasive cutaneous melanoma in our hospital between January 1, 2007, through December 31, 2016. We excluded those with just in situ melanoma or a primary report of metastasis of melanoma. The study population consisted of 163,100 members of the HMCP.

We found 253 cutaneous malignant melanoma (CMM) cases, witch 124 were females (49.0%). The median age at diagnosis was 69 years (IQR 58–78), and the average age at diagnosis was 66.3 years (SD=15.3). This result is in line with the higher incidence of CMM, as well as with other types of cancer, in older people. The average age of diagnosis for CMM worldwide is 57, while our data show a mean age of 66.^5^

The HCMP crude CMM incidence density rate (IDR) obtained was 19.5 per 100,000 person-years (95% CI 16.3–21.0). The adjusted IDR for the BA population was 13.4 per 100,000 person-years (95% CI 11.7–15.2). According to 2010 national demographic census data, BA has a population of 2,890,151 inhabitants, of which 30% are <25 years and 21.7% are >60 years. The distribution over age and gender strata are similar to the HMCP population (Fig. 1 a and b). The adjusted IDR for CMM for the population in Argentina is 10.2 per 100,000 person-years (95% CI 8.7–11.7), almost half of the crude HCMP IDR. This adjustment-based discrepancy can be attributed to the difference in the age distribution within the population under study, in which 32% of the members are over the age of 60 compared to the population of Argentina, which only has 10.2% belonging to the same age group.

**Figure 1:**
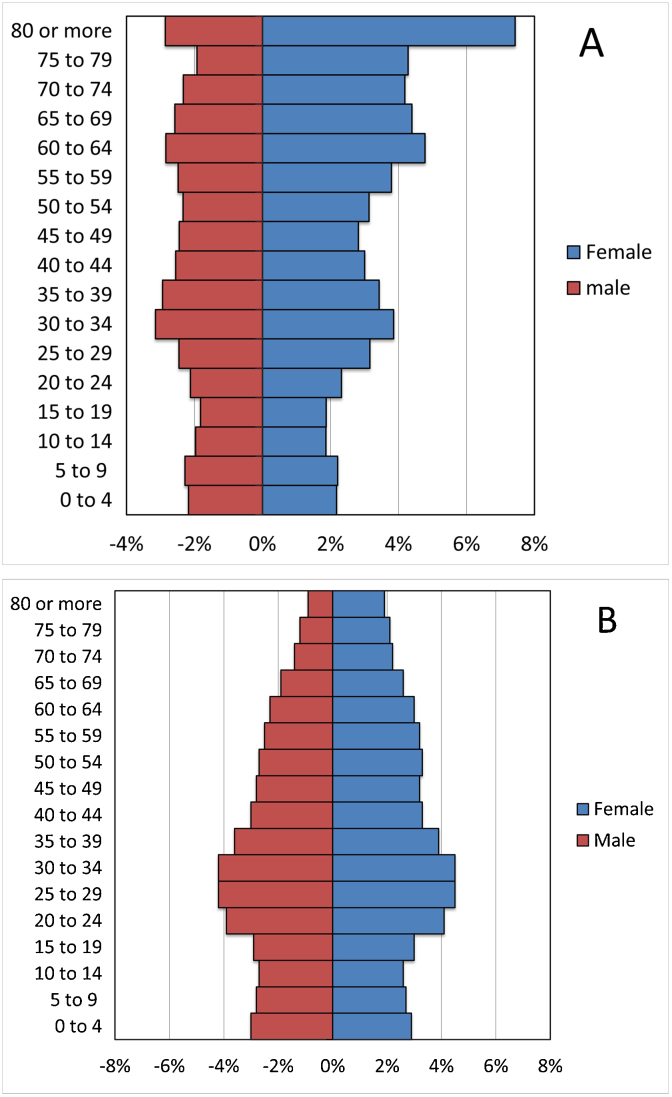
Gender and age distribution of (A), Italian Hospital Medical Care Program and (B), Buenos Aires, population according to 2010 national demographic census. Male (red) Female (blue). The age of the population is grouped into periods of four years. The quantities of people of each gender are expressed in percentage.

Prevalence was estimated considering the number of live cases up to July 1, 2016, divided by the number of active HCMP members at that time. The prevalent cases were 193 out of 146,524 HCMP members with a prevalence rate of 13.2 per 100,000 people (95% CI 11.4–15.2).

When we analyze rates by gender, the literature says that in young people the incidence of CMM is higher in women. It is inverted in older people, with the higher incidence being in men.^6^ In our population, we found a similar situation, HCMP gender-specific crude IDRs were 23.6 per 100,000 person-years (95% CI 18.7–26.8) for males and 15.9 per 100,000 person-years (95% CI 13.1–18.8) for females. The adjusted IDR for the BA population was 16.3 per 100,000 person-years (95% CI 12.4–20.2) and 12.3 per 100,000 person-years (95% CI 9.9–14.6), respectively (Table 1).

**Table 1:** Crude and adjusted IDR. Values in Cases per 100,000 person-year and (95% CI) are presented by region. Adjusted to Buenos Aires and Argentina population according to 2010 demographic census.

	Crude IDR	Adjusted IDR to Buenos Aires	Adjusted IDR to Argentina	Adjusted IDR to WHO
**All CMM**	19.5 (16.3–21.0)	13.4 (11.6–15.2)	10.2 (8.7–11.7)	9.6 (8.1–11.1)
**Male CMM**	23.6 (18.7–26.8)	16.3 (12.4–20.2)	13.2 (8.6–17.7)	6.5 (4.3–8.8)
**Female CMM**	15.9 (13.1–18.8)	12.3 (9.9–14.6)	9.6 (7.6–11.5)	4.4 (3.5–5.4)

The incidence rate ratio (IRR) of the crude IDR for males compared to females was 1.5 (95% CI 1.2–1.9; p = 0.0017). This data suggests a higher probability of developing this type of tumor in men. This is probably due to fewer prevention strategies since, in our country, men were the ones primarily exposed to the sun for work reasons in past decades. In the meantime, the population of women has started increasing in this regard due to tanning beds and intermittent sun exposure for recreational or beauty purposes.^2^, ^7^

On a worldwide level, we find a wide range of rates for CMM. The annual incidence ranges from 0.3 and 0.2 per 100,000 person-years in Asia and India, respectively, up to 55 per 100,000 person-years in New Zealand, a position held by Australia not long ago, which has recently dropped to 54 per 100,000 person-years.^7^, ^8^ The most recent publications are from Germany and Canada, where the crude IDR observed for the latter were 12.3 cases per 100,000 person-years and the ASIR (age-standardized incidence rate) for the population was 9.6 cases per 100,000 person-years. According to these publications, our adjusted IDR for CMM is similar to the German and Canadian populations.^9^ In South America, Brazil and Colombia have recently published data on this matter. In 2016, Brazil had an estimated annual melanoma incidence of 5.8 cases per 100,000 person-years.^6^, ^8^, ^10^ Colombia had an ASIR of 1.7 per 100,000 person-years. Both Brazil and Colombia have a greater proportion of populations of African descent compared to Argentina. This could explain the differences in the rates of the CMM.

Since this is a review, we had several limitations. The validity of this study could be increased with the addition of information from other institutions from BA and representative regions nationwide to confirm the results and achieve a more accurate knowledge of melanoma epidemiology in Argentina. However, this is the first study of a large cohort of patients in Argentina that estimated the incidence of melanoma, and these results are similar to those published for other similar geographical areas.

## Financial support

None declared.

## Authors’ contributions

Adriana Raquel Rinflerch: Conception and design of the study, analysis, and interpretation of data; drafting the article and revising it critically for important intellectual content; final approval of the version to be submitted.

Victoria Ines Volonteri: Conception and design of the study; draft the article and revising it critically for important intellectual content; final approval of the version to be submitted.

María Cecilia Roude: Acquisition of data; draft the article and revising it critically for important intellectual content; final approval of the version to be submitted.

Vanina Laura Pagotto: Analysis and interpretation of data; draft the article and revising it critically for important intellectual content; final approval of the version to be submitted.

Melina Pol: Acquisition of data; draft the article and revising it critically for important intellectual content; final approval of the version to be submitted.

Luis Daniel Mazzuoccolo: The conception and design of the study.: draft the article and revising it critically for important intellectual content; final approval of the version to be submitted.

## Conflicts of interest

None declared.

## Acknowledgement

This work was funded by grants from the 10.13039/100000054National Cancer Institute (INC 2018), and the HIBA Dermatology Service.
